# Metastatic extraneural glioblastoma diagnosed with molecular testing

**DOI:** 10.1093/oncolo/oyae115

**Published:** 2024-06-05

**Authors:** Nazanin K Majd, Henry Hiep Vo, Cesar A Moran, Shiao-Pei Weathers, I-Wen Song, Garret L Williford, Jordi Rodon, Siqing Fu, Apostolia-Maria Tsimberidou

**Affiliations:** Department of Neuro-Oncology, The University of Texas MD Anderson Cancer Center, Houston, TX, United States; Department of Investigational Cancer Therapeutics, The University of Texas MD Anderson Cancer Center, Houston, TX, United States; Department of Pathology, The University of Texas MD Anderson Cancer Center, Houston, TX 77030, United States; Department of Neuro-Oncology, The University of Texas MD Anderson Cancer Center, Houston, TX, United States; Department of Investigational Cancer Therapeutics, The University of Texas MD Anderson Cancer Center, Houston, TX, United States; Department of Neuro-Oncology, The University of Texas MD Anderson Cancer Center, Houston, TX, United States; Department of Investigational Cancer Therapeutics, The University of Texas MD Anderson Cancer Center, Houston, TX, United States; Department of Investigational Cancer Therapeutics, The University of Texas MD Anderson Cancer Center, Houston, TX, United States; Department of Investigational Cancer Therapeutics, The University of Texas MD Anderson Cancer Center, Houston, TX, United States

**Keywords:** glioblastoma, extraneural metastases, next-generation sequencing, brain tumor

## Abstract

Glioblastoma, the most common malignant brain tumor in adults, is associated with a median overall survival duration of less than 2 years. Extraneural metastases occur in less than 1% of all patients with glioblastoma. The mechanism of extraneural metastasis is unclear. We present a case of extensive extraneural, extraosseous, epidural, and soft-tissue metastasis of glioblastoma. The diagnosis of metastatic glioblastoma was made only after next-generation sequencing (NGS) of the metastatic paraspinal lesions was completed. The *CDK4, pTERT, PTEN,* and *TP53* molecular alterations seen in the initial intracranial glioblastoma were found in the paraspinal tumor, along with the addition of *MYC,* which is implicated in angiogenesis and epidermal-to-mesenchymal transition. Immunohistochemical stains showed that neoplastic cells were negative for GFAP. In conclusion, this case raises awareness about the role of NGS in the diagnosis of extraneural glioblastoma. This diagnosis was not possible with histology alone and only became evident after molecular profiling of the metastatic lesions and its comparison to the original tumor.

## Teaser

This case raises awareness about the role of next-generation sequencing in the diagnosis of extraneural glioblastoma.

**Implications for practice**

Extraneural metastases in glioblastoma are rare and challenging to diagnose based on histology alone, and the underlying molecular mechanisms of metastasis are unclear. Here, we describe a case where a diagnosis of metastatic glioblastoma only became evident after molecular profiling of the metastatic lesion and its comparison to the original tumor, highlighting the importance of next-generation sequencing for prompt and accurate diagnosis, which can aid treatment decisions and prognosis discussions. We also demonstrated gained molecular alterations in the metastatic lesion in comparison with the original tumor, which provides insights in understanding the pathogenesis and evolution of intracranial glioblastoma from the initial tumor to the metastatic site.

## Introduction

Glioblastoma, the most common malignant brain tumor in adults, is associated with a median overall survival duration of less than 2 years.^1^ Among patients with glioblastoma, extraneural metastases are very rare and comprise less than 1% of all cases.^2^ It is postulated that the incidence of extraneural metastasis is low because the microenvironment outside of the central nervous system (CNS) is inadequate for glioma cell growth and/or because the survival of patients with glioblastoma is dismal. The most common extraneural metastatic sites are the lungs, lymph nodes, and bones.^3,4^ The pathogenesis of extraneural metastasis is unclear, but potential mechanisms include direct invasion through dura and bone, venous invasion *via* the leptomeningeal sinuses or intracerebral veins, and lymphatic drainage of cerebrospinal fluid into the extraneural tissue.^5^ Even though molecular markers of poor prognosis of intracranial glioblastoma such as isocitrate dehydrogenase (*IDH*) wild-type and methyl-guanine methyl transferase (MGMT) promoter unmethylated status have been identified, data about the acquired molecular changes that allow the tumor to grow outside of the nervous system are limited. Metastatic lesions are rarely biopsied and profiled molecularly; however, molecular analysis is necessary to confirm the diagnosis of metastatic glioblastoma by ruling out other primary neoplastic processes.

Here, we present a case of extensive extraneural, extraosseous, epidural, and soft-tissue metastasis of glioblastoma, which posed a diagnostic challenge because of its atypical imaging and histological features. The diagnosis of metastatic glioblastoma was made only after next-generation sequencing (NGS) of the metastatic paraspinal lesions showed that they shared molecular alterations with the original intracranial tumor, indicating clonal evolution in the extraneural metastases. This unique case of molecularly defined metastatic glioblastoma highlights the challenges involved in establishing a histologic diagnosis and demonstrates the evolution of molecular aberrations noted in the metastatic foci.

## Methods

### Intracranial tumor

The MD Anderson Solid Tumor Genomic Assay (STGA) was used to analyze the newly diagnosed intracranial tumor: NGS-based analysis included gene panel mutations in the coding sequence of 134 genes and selected amplifications in 47 genes (overlap: 146 genes total) in our CLIA-certified molecular diagnostics laboratory.

### Metastatic lesion

The tissue obtained from the paraspinal biopsy was fixed in 10% buffered formalin and embedded in paraffin. Sections were obtained from the paraffin-embedded tissue and stained with hematoxylin and eosin (H&E). From the same paraffin block, unstained sections were obtained to perform an extensive panel of immunohistochemical stains that included epithelial markers (pan-keratin, keratin 7, keratin 20, low-molecular-weight keratin, CAM 5.2, and p40), neuroendocrine markers (synaptophysin and chromogranin), neural markers (S-100 protein and GFAP) mesenchymal markers (desmin, myogenin, Myo-D), vascular markers (CD34, Erg), and other more specialized markers for lymphoid and other epithelial tumors of specific organs (BAF47, cyclin D1, CD99, TTF-1, CD5, CD3, CD20, PAX5, CD45RB, NKX3.1, PSA, PSMA, NUT1, Ki-67). All these markers were run with their respective dilutions and negative controls.

In addition, unstained sections were obtained to perform mutation analysis precision panel (MAPP) assays with a custom high-throughput next-generation sequencing-based CLIA assay that targeted hybridization-based capture technology for detection of sequence variants/mutations in 610 genes, copy number variants in 583 genes, selected gene rearrangement in 34 genes, and selected genomic immune-oncology signatures including microsatellite instability and tumor mutational burden. The MAPP assay uses the NovaSeq 6000 next-generation sequencing platform and bidirectional paired-end sequencing to identify nucleic acid variants for all coding regions from most genes in the panel. Reported somatic mutations are identified by comparison to the human genome reference sequence GRCh37/hg19 and reviewed in OncoSeek against a process-matched normal control.

## Results

**Figure 1.**
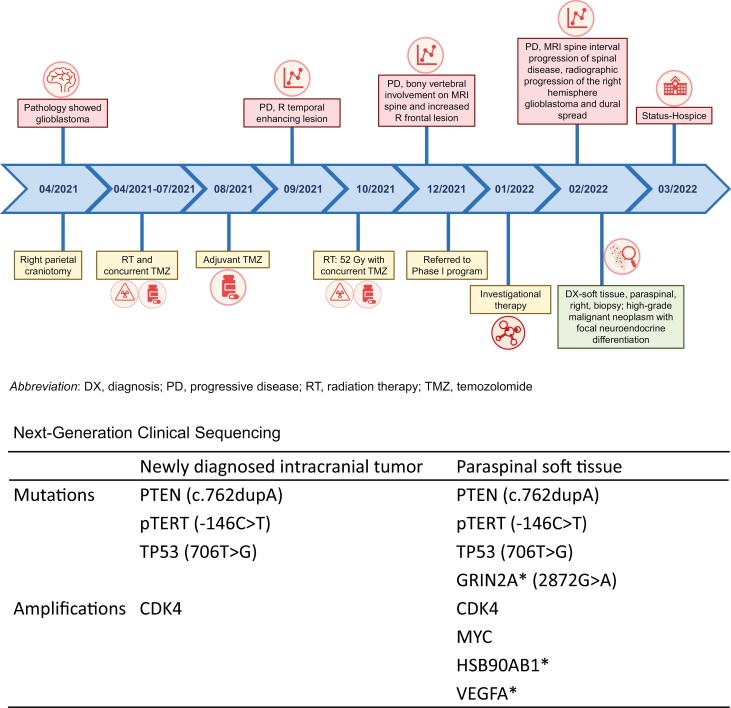
Patient clinical history and next-generation sequencing of the intracranial glioblastoma tumors and metastatic lesion.

**Figure 2.**
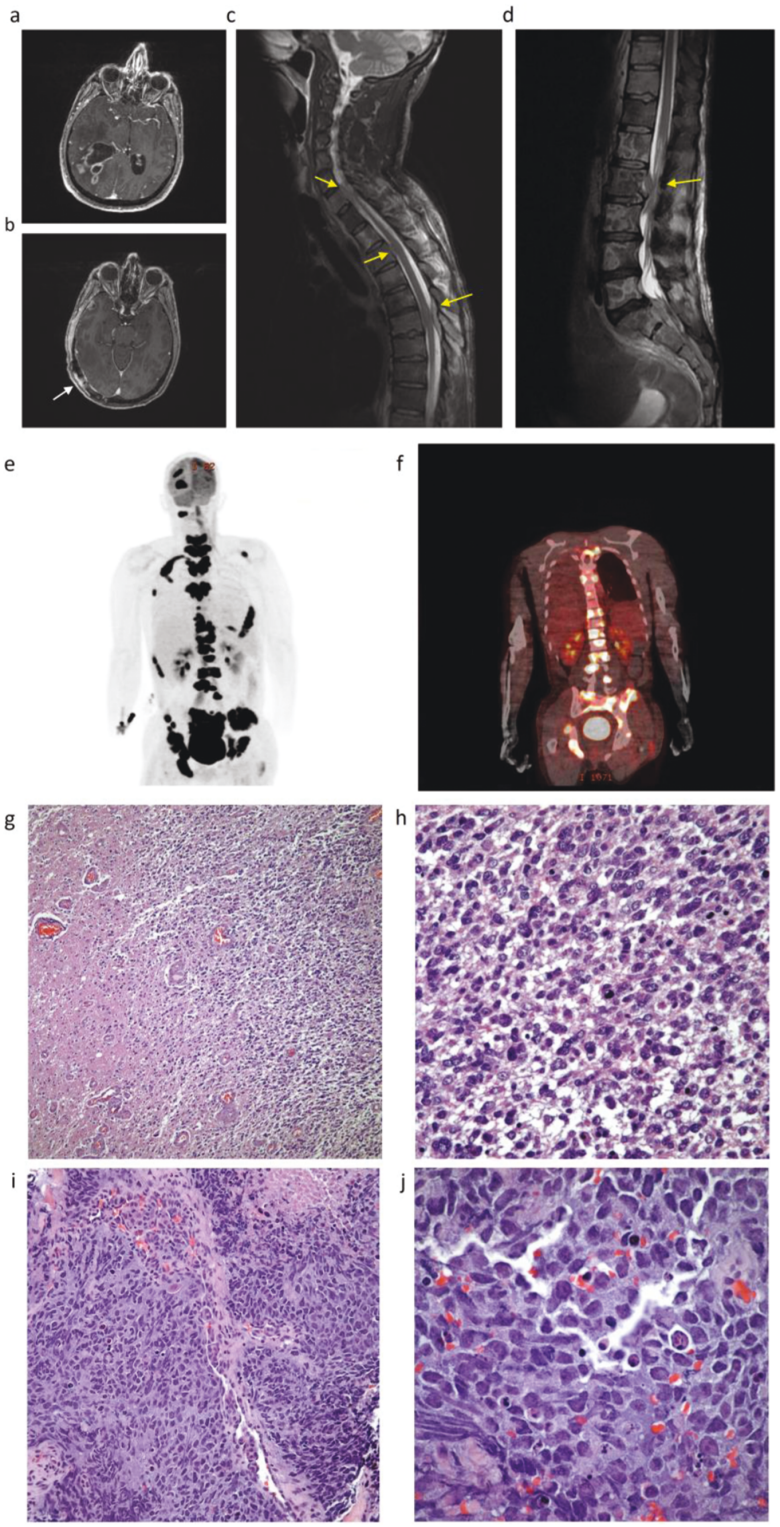
a. MRI T1 post-contrast axial image demonstrates a large, heterogeneously enhancing cystic lesion in the right parietal lobe; b. MRI T1 post-contrast axial image demonstrates an enhancing lesion in the right temporal lobe outside of the initial radiation field (white arrow); c-d. MRI of the cervical and thoracic spine and lumbosacral image demonstrates extensive extraosseous epidural and soft-tissue metastasis. Examples are shown with an arrow at the level of C6, T2, T5-T6, and L2; e-f. PET/CT demonstrates numerous sites of abnormal FDG avidity within the skeleton, extending into the adjacent soft-tissues and the spinal canal. g-h. H&E staining of initial intracranial glioblastoma: g. Neoplastic cellular proliferation replacing normal brain. Note the presence of relatively normal brain matter on the left side (H&E 10×). h. Higher magnification shows cells with round to oval nuclei, moderate amounts of eosinophilic cytoplasm, and several mitotic figures—all features of grade 4 glioblastoma (H&E 40×). i-j. H&E staining of extraneural paraspinal metastatic glioblastoma: i. Poorly differentiated malignant neoplasm with focal areas of necrosis (H&E 20X); j. Tumor is composed of large cells with indistinct cell borders, round to oval nuclei, and nucleoli. Mitotic figures are present (H&E 60×).

An overview of the patient’s clinical history is depicted in Figure 1. A right-handed man in his late 40s presented with new-onset progressive headaches. Magnetic resonance imaging (MRI) of the brain demonstrated a large, heterogeneously enhancing cystic lesion in the right parietal lobe (Figure 2a). He underwent gross total resection of the lesion, and the pathology findings were consistent with glioblastoma. NGS sequencing using STGA identified *CDK4* amplification and *pTERT*, *PTEN*, and *TP53* mutations (Figure 1). The tumor was *IDH* wild-type and the MGMT promoter region was unmethylated. The patient’s postoperative neurological examination was unremarkable, except for left homonymous hemianopia. His Karnofsky performance scale score was 90. He underwent standard-of-care radiation therapy (60 Gy, 30 fractions) and concurrent temozolomide, which he tolerated well.

The patient’s post-chemoradiation therapy MRI showed subtle enhancement surrounding the surgical cavity. After completion of one cycle of adjuvant temozolomide, MRI of the brain demonstrated a new enhancing lesion in the right temporal lobe outside of the initial radiation field, and the right parietal resection cavity was stable (Figure 2b). The patient received radiation therapy (52 Gy, 15 fractions) and concurrent temozolomide for the right temporal lesion.

Three weeks after completion of radiation therapy, the patient presented with back pain. Computed tomography (CT) of the chest, abdomen, and pelvis with contrast and CT of the thoracic and lumbar spine without contrast were unremarkable, except for mild degenerative disc disease of the thoracic and lumbar spine. Two weeks later, an MRI of the spine showed diffuse skeletal lesions with paraspinal and epidural tumor extension throughout the cervical, thoracic, and lumbar spine and pelvis and enhancement of the nerve roots of the cauda equina. MRI of the brain showed multifocal areas of dural/extra-axial disease in the right frontal and temporal lobes. The patient was started on a clinical trial with a CDK4 inhibitor. On cycle 1, day 21, he was admitted to the hospital with worsening back pain. A neurological examination showed no significant changes since the last assessment, 3 weeks earlier. MRI of the spine showed extensive extraosseous epidural and soft-tissue lesions (Figure 2c, d). Positron emission tomography (PET)/CT imaging showed numerous sites of abnormal fluorodeoxyglucose (FDG) avidity within the skeleton, extending into the adjacent soft-tissues and the spinal canal (Figure 2e, f).

Given the unusual findings for glioblastoma and the findings on PET/CT, lymphoproliferative disorders were considered. A paraspinal core needle biopsy demonstrated benign skeletal muscle with focal atrophy and hemorrhage and was non-diagnostic. Bone marrow biopsy showed a high-grade, poorly differentiated malignant neoplasm involving ~80% of the medullary space (bone marrow differential: 56% tumor cells; inadequate tissue for cytogenetic analysis; flow cytometry, negative for aberrant B- or T cells). Another paraspinal biopsy demonstrated a high-grade malignant neoplasm with focal neuroendocrine differentiation (not further classified), and a Ki-67 index of 80% (Figure 2c, d, i). Immunohistochemical stains showed that neoplastic cells were immunoreactive for p53, synaptophysin (focal), chromogranin (focal), cyclin D1 (subset), BAF47 (retained nuclear expression), while immunonegative for TTF-1, pancytokeratin, CK7, CK 20, CAM 5.2, CD34, CD 99, CD5, CD3, CD20, PAX 5, CD45RB, NKX3.1, androgen receptor, PSA, PSMA, ERG, NUT1, p40, GFAP, S100, Myogenin, Myo-D1, and Desmin. H&E staining of the initial intracranial glioblastoma and of the extraneural paraspinal metastatic glioblastoma is shown in Figure 2g, h and Figure 2i, j, respectively.

To further characterize the neoplasm, NGS of tumor tissue obtained from the second paraspinal biopsy was initiated using The MD Anderson Mutation Analysis Precision Panel (MDA MAPP). The *CDK4* amplification and *pTERT, PTEN, and P53* mutations seen in the initial intracranial glioblastoma were found in the paraspinal tumor, along with *MYC* amplification. Additional molecular aberrations found on the metastatic foci obtained on MDA MAPP were *HSP90AB1* and vascular endothelial growth factor (*VEGF*)-A amplifications and *GRIN2A* mutation (Figure 1). It is notable that *MYC* was included in the analysis of both intracranial and metastatic lesions, but *HSP90AB1*, *VEGFA,* and *GRIN2A* were not included in the NGS panel used for the analysis of the intracranial tumor. While awaiting the final results of molecular testing (10 months after the initial diagnosis of glioblastoma), the patient developed progressive neurological decline and paraparesis that quickly progressed to paraplegia despite high-dose steroids. Palliative radiation therapy was attempted but aborted owing to poor tolerance. The patient was transitioned to hospice care and passed away 1 month later.

## Discussion

Here, we present a rare case of glioblastoma with rapid clinical deterioration that required NGS analysis to make the diagnosis of extraneural metastases. Histologic and extensive immunohistochemical testing of the extraneural tumor, which was biopsied twice, and the bone marrow were insufficient to classify the metastatic tumor. Identification of the molecular alterations seen in the original tumor that included *CDK4* amplification and *pTERT, PTEN,* and *P53* mutations confirmed the diagnosis of extraneural glioblastoma.

In addition to these molecular alterations, amplification of *MYC, HSP90AB1, and VEGF*-A and mutation of *GRIN2A* were present in the metastatic tumors. While *MYC* amplification was an acquired molecular event (included in the NGS panel used for the initial and metastatic tumor), it is not clear whether *HSP90AB1, VEGFA, or GRIN2A* were present at the time of initial diagnosis. Nevertheless, the 3 gene amplifications in the metastatic tumors are implicated in angiogenesis and epidermal–mesenchymal transition. The *MYC* oncogene encodes a transcription factor that regulates several genes related to cell cycle progression, differentiation, and cellular transformation.^6^*MYC* amplification is noted in 0.88% of patients with glioblastoma,^7^ and it is the most frequently amplified gene across all cancer types, occurring in 21% of cases based on recent cancer genome atlas analysis.^8^*MYC* enhances the self-renewal capacity of glioblastoma stem-like cells and maintains their tumorigenic potential.^9,10^ In addition, overexpression of *MYC* has been shown to induce epithelial–mesenchymal transition, angiogenesis, and tumorigenesis in mammary epithelial cells.^11,12^*HSP90AB1* (heat shock protein 90 kDA, alpha, class B, member 1) is a member of the large family of HSP proteins, which are molecular chaperones that enable proper folding, refolding, and stabilization of vital proteins within the cell. *HSP90AB1* alterations are found in approximately 1% of all cancers, with the highest prevalence in glioblastoma, followed by colorectal adenocarcinoma and lung adenocarcinoma.^7^*HSP90AB1* stabilizes *VEGF-A*-induced pro-angiogenic protein *BAZF* (BCL-6 associated zinc finger protein) and may positively regulate angiogenesis.^13^ The role of *HSP90AB1* in glioblastoma remains unclear. *VEGF*-*A*—the prototype member of the *VEGF* family—predominantly regulates the process of angiogenesis in the CNS and directly interacts with *VEGF* receptors expressed on cancer cells, stimulating disease progression.^14^*VEGF* overexpression is an adverse prognostic factor in many cancers, including glioblastoma.^15^ Lastly, mutations in GRIN2A, which encodes the NMDA receptor subunit NR2A, have been associated with poor survival in melanoma,^16^ but its role in glioblastoma remains unclear. Although the role of these alterations in the aggressive nature of patient’s tumor is unclear, it is plausible that they are contributing factors. We have previously reported a partial response that lasted for 10 years in a patient with glioblastoma with *CDK4* amplification and *FGFR3-TACC3* chromosomal fusion, treated with bevacizumab and valproic acid, that targeted multiple pathways associated with tumor progression and angiogenesis.^17^

Other investigators have reviewed 28 published cases of glioblastoma metastatic to the vertebra.^18^ They reported that the mean age at presentation was 38.4 years and the average overall survival was 26 months. Interestingly, the clinical presentation ranged from asymptomatic disease to varying degrees of pain, extremity weakness, or other neurologic deficits. In 8 (28.6%) patients, the diagnosis was made via autopsy. The median survival duration after diagnosis of vertebral metastasis was 10 months.^18^

In another series of 10 patients seen at the Memorial Sloan Kettering Cancer Center with extraneural metastases of glioblastoma (9 patients) and gliosarcoma (1 patient), the median age was 39 years; 7 patients were men and 3 women.^19^ All patients had surgical resection and radiation therapy; and 9 patients received temozolomide, with subsequent individualized chemotherapy. The median overall survival from initial diagnosis was 19.6 months (range 11.2-57.5 months) and from extraneural metastasis, 5 months (range 1-16.1 months). The most common genomic alterations identified in 8 patients were *P53* (*n* = 5), *RB1* (*n* = 5), *PTEN* (*n* = 4), *TERT* (*n* = 4), *ATRX* (*n* = 4), *NF1* (*n* = 3), *IDH1* (*n* = 1), *EGFR* amplification (*n* = 2), and *EGFR* mutation (*n* = 1). In 3 of these 5 cases, the *TP53* mutation was found in both the primary and metastatic sites. *ATRX*, *PTEN*, *RB1*, *TERT*, *IDH1*, and *NF1* were found in both primary and metastatic sites; *TP53* and *RB1* were both mutated in 4 out of 7 (57%) sequenced primary tumors. The authors concluded that several risk factors emerged for extraneural metastasis of glioblastoma and gliosarcoma, including sarcomatous dedifferentiation, disruption of normal anatomic barriers during surgical resection, and tumor suppressor gene alterations.^19^

## Conclusions

Our case raises awareness regarding the diagnosis of extraneural glioblastoma and highlights the important role of molecular analysis in the diagnosis of extraosseous, epidural, or soft-tissue metastases of glioblastoma. The mechanism of metastasis for diffuse gliomas is not well-understood, in part due to the rarity of these cases. In our case, next-generation sequencing provided insights into understanding the pathogenesis and evolution of intracranial glioblastoma from the initial tumor to the metastatic site. The widespread use of next-generation sequencing as a diagnostic tool has the potential to shed light on the acquired molecular aberrations that drive extraneural metastasis in glioblastoma.

## Data Availability

Sequencing data are not publicly available in order to protect patient privacy. The ethics committee does not allow for these data to be deposited into a secure access, controlled repository. Qualified researchers can apply for access to the data by contacting the corresponding authors at NKMajd@mdanderson.org and atsimber@mdanderson.org. The data will be made available on reasonable request.
